# Implications of the strain irreversibility cliff on the fabrication of particle-accelerator magnets made of restacked-rod-process Nb_3_Sn wires

**DOI:** 10.1038/s41598-019-41817-7

**Published:** 2019-04-02

**Authors:** Najib Cheggour, Theodore C. Stauffer, William Starch, Loren F. Goodrich, Jolene D. Splett

**Affiliations:** 1 Department of Physics, University of Colorado, Boulder, CO 80309 USA; 2 Quantum Electromagnetics Division, National Institute of Standards and Technology, Boulder, CO 80305 USA; 3 Applied Superconductivity Center, National High Magnetic Field Laboratory, Florida State University, Tallahassee, FL 32310 USA; 4 Statistical Engineering Division, National Institute of Standards and Technology, Boulder, CO 80305 USA

**Keywords:** Superconducting properties and materials, Applied physics, Characterization and analytical techniques

## Abstract

The strain irreversibility cliff (SIC), marking the abrupt change of the intrinsic irreversible strain limit *ε*_irr,0_ as a function of heat-treatment (HT) temperature *θ* in Nb_3_Sn superconducting wires made by the restacked-rod process (RRP^®^), is confirmed in various wire designs. It adds to the complexity of reconciling conflicting requirements on conductors for fabricating magnets. Those intended for the high-luminosity upgrade of the Large Hardon Collider (LHC) at the European Organization for Nuclear Research (CERN) facility require maintaining the residual resistivity ratio *RRR* of conductors above 150 to ensure stability of magnets against quenching. This benchmark may compromise the conductors’ mechanical integrity if their *ε*_irr,0_ is within or at the bottom of SIC. In this coupled investigation of strain and *RRR* properties to fully assess the implications of SIC, we introduce an electro-mechanical stability criterion that takes into account both aspects. For standard-Sn billets, this requires a strikingly narrow HT temperature window that is impractical. On the other hand, reduced-Sn billets offer a significantly wider choice of *θ*, not only for ensuring that *ε*_irr,0_ is located at the SIC plateau while *RRR* ≥ 150, but also for containing the strain-induced irreversible degradation of the conductor’s critical-current beyond *ε*_irr,0_. This study suggests that HT of LHC magnets, made of reduced-Sn wires having a Nb/Sn ratio of 3.6 and 108/127 restacking architecture, be operated at *θ* in the range of 680 to 695 °C (when the dwell time is 48 hours).

## Introduction

The expected application of Nb_3_Sn superconducting wires in the high-luminosity (HL) upgrade of the Large Hadron Collider (LHC) at the European Organization for Nuclear Research (CERN) facility is dictated by the need for magnetic-field intensities in the range of 11 to 13 T, required to increase the rate of particle collisions at the ATLAS (A Toroidal LHC ApparatuS) and CMS (Compact Muon Solenoid) detectors of the LHC^1,2^. Such intensities are beyond the practical limits of Nb-Ti, which has been the main conductor for particle-accelerator technologies to date^3–5^. Tens of Nb_3_Sn dipoles and quadrupoles with large apertures will be constructed to replace the Nb-Ti magnets in the collimation system and beams’ interaction regions. The upgrade project (HL-LHC) will consume about 30 tonnes of state-of-the art, very high critical-current density *J*_c_, Nb_3_Sn wires^2^, and will be a crucial step for testing the maturity of Nb_3_Sn technology for its potential usage to extend the field range to 16 T in future high-energy particle colliders^1^. (In this paper, wire is interchangeably referred to as conductor, billet, or strand).

The internal-tin restacked-rod process RRP^®^ for fabricating Nb_3_Sn wires increases the cross-sectional area of the conductor’s superconducting component and promotes a homogeneous diffusion of Sn to Nb rods during the heat-treatment (HT) reaction for forming the A15 phase^6–8^. These improvements are achieved through increasing the fractions of Sn and Nb and decreasing that of Cu in the wire design^6–8^. It results in Nb_3_Sn occupying about a third of the wire’s cross-section^9^, with a near stoichiometric composition^6–9^. Both of these features are needed to obtain very high *J*_c_. On the other hand, during HT, individual filaments coalesce into one solid Nb_3_Sn tube inside each of the wire sub-elements, of a diameter in the range of 35 to 100 μm depending on the wire architecture^7,8^.

Whereas *J*_c_ values in excess of 3,000 A/mm^2^ at 12 T and 4.2 K obtained in RRP Nb_3_Sn wires are sufficient for HL-LHC, magneto-electrical instabilities that stem from magnetic-flux and electric-current fast redistributions in the conductor during current ramping could be challenging^10–18^. These instabilities, referred to as magnetization and self-field instabilities, respectively, generate local heat and temperature rise that can cause magnets to quench at currents significantly below the conductor’s critical surface of *J*_c_ versus magnetic field *B* at low and moderate field ranges^10–18^. Reducing the size of Nb_3_Sn sub-elements can mitigate these instabilities significantly, and a sub-element size around 20 μm or less is desirable^1^. However, size reductions below 50 μm in RRP wires have resulted in a dramatic drop of *J*_c_^8,19^. In addition, such wires still need further development to become technological conductors that can be readily fabricated in piece-lengths sufficient for applications. Furthermore, such sub-element reduction may impose additional limitations on HT temperature and duration to avoid breaching distributed Nb barriers that would otherwise result in a significant decrease of the wire’s residual resistivity ratio *RRR*^1^, a parameter that is also important to remedy conductor instabilities^11–14,16,17^.

Magnetization and self-field instabilities may be contained if *RRR* of the conductor’s Cu component is sufficiently high^14,16,17^. Basically, magnet quenching can be prevented if disturbance-generated heat is evacuated to the surrounding helium bath fast enough through Cu. The thermal conductivity of Cu improves by a factor of about 3.4, in the temperature range 1–10 K, when its *RRR* is increased from 30 to 100^20^. Such an improvement may be useful for a more effective evacuation of heat away from the magnet^21^. Extensive empirical and simulation studies of wire instabilities suggested that *RRR* be at least 100 in the final conductor form (once cabled and reacted as part of the magnet) to mitigate instability effects^14,16,17^. This translates to an *RRR* in undeformed conductor (i.e. before cabling it) of at least 150.

This requirement RRR ≥ 150 was achieved, first, by applying less aggressive HTs that promote *RRR* but at the expense of *J*_c_^14,16,17,22^. Thereafter, a 6% reduction of the amount of Sn in the wire turned out to be very effective for meeting the *RRR* requirement^8^. Indeed, a change of the ratio Nb/Sn from 3.4 in early RRP billets (referred to as standard-Sn billets) to 3.6 in new ones (reduced-Sn billets) was a beneficial adjustment in the wire design^8^. Even though *J*_c_ of reduced-Sn billets decreases due to the reduction of the amount of Sn in the wire, it can be recovered partially by increasing the HT temperature typically from 640–650 °C for the standard-Sn billets to 665–680 °C for the reduced-Sn billets without compromising the *RRR* benchmark.

The sub-element size for the wires to be fabricated for HL-LHC has been set to a compromise value of about 55 μm to maintain a sufficiently high and homogeneous *J*_c_^2,23^, further underscoring the need to maintain high *RRR* to mitigate wire instabilities.

Another aspect of great importance is the inherent high sensitivity of Nb_3_Sn superconducting properties to strain, unlike the ductile Nb-Ti material (see for example^24–37^ and references therein). These properties also degrade irreversibly when mechanical cracks start to form in Nb_3_Sn under the effects of stress and strain (see for example^38–46^ and references therein). By incorporating the brittle Nb_3_Sn superconductor in a particle-accelerator machine, where mechanical forces on the conductor are going to be intense, especially at high magnet fields, strain and stress limits for conductor handling and operation naturally come into play in the design, fabrication, transportation, and cool-down and powering of accelerator magnets^47–53^. Both axial and transverse strain components should be taken into account^54–56^. In this paper, we will discuss the axial strain limits and irreversible effects.

In a recent report, the U.S. high-luminosity LHC accelerator upgrade project (US HL-LHC AUP) added a requirement “*ε*_irr,0_ > 0.25%” to the strand design criteria for the fabrication of quadrupole magnets^23^, *ε*_irr,0_ being the intrinsic irreversible axial-strain limit where irreversible effects first appear in the behavior of the conductor’s transport critical current *I*_c_ as a function of axial strain *ε* to which it is subjected. The benchmark value of 0.25% is actually empirical, based on earlier strain characterizations of Ti-doped RRP Nb_3_Sn wires^41^. In effect, even higher *ε*_irr,0_ values are better to help reduce the complexity of the mechanical structures and tooling needed for stress and strain management in magnets.

We recently showed that *ε*_irr,0_ depends strongly on HT conditions in RRP Nb_3_Sn standard-Sn billets either doped with Ti or Ta. We found that *ε*_irr,0_ undergoes a precipitous and large change as a function of HT temperature *θ*, called the strain irreversibility cliff (SIC)^46^. As we discussed in^46^ and will show in great detail herein, SIC too imposes certain conditions on HT with competing requirements vis-à-vis those favorable to *RRR*. As such, SIC adds to the complexity of the trade-offs among *J*_c_, *RRR*, and sub-element size benchmarks. Therefore, studies of the conductors’ electromechanical properties should be combined with investigations of *J*_c_ and *RRR* as a function of conductor design and HT to find optimal parameters for magnet conditioning. In order to evaluate the practical implications of SIC comprehensively, such approach is essential.

In this paper, we build on our previous strain characterization of standard-Sn billets RRP 13711-2 (Ta-doped) and 11976-1 (Ti-doped) by adding two more billets, RRP 14943-2a and 14984, both Ti-doped (Table 1). Billet 14984 has a reduced-Sn content. We will put more emphasis on it as it is a pre-production billet for HL-LHC. We investigate if such a conductor exhibits the SIC behavior and how it is affected by the Sn content. Billet 14943-2a is a standard-Sn, used for comparisons and for gaging reproducibility of the results with respect to billet 11976-1 that has the same design but a slightly smaller diameter (Table 1). Furthermore, we expand the study to encompass *RRR* measurements on all four billets. We investigate how the HT temperature *θ* affects *ε*_irr,0_ (and SIC), *I*_c_, and *RRR* of a given conductor, and define the range of *θ* that is suitable to fulfil the competing requirements on the multiple parameters for each of the conductors and the role played by the content of Sn and the doping element.

**Table 1:** Design characteristics of the RRP Nb_3_Sn wires investigated, all having 108/127 restacking architecture.

Billet number	Wire diameter after reaction (mm)	Wire twist pitch (mm)	Non-Cu (%)	Subelement size (μm)	Nb/Sn Ratio	Dopant (nominal content)
13711-2	0.82	13	46.5	52	3.4 (standard-Sn)	Ta (4 at. %)
11976-1	0.82	12	48.9	52	3.4 (standard-Sn)	Ti (1.5 at. %)
14943-2a	0.87	16	46.5	55	3.4 (standard-Sn)	Ti (1.5 at. %)
14984	0.87	19	46.5	55	3.6 (reduced-Sn)	Ti (1.5 at. %)

Each of the four billets has 108 Nb_3_Sn sub-elements distributed around 19 Cu sub-elements at the billet center (design 108/127). Additional details are provided in Table 1. The HT (made in vacuum) consists of two pre-stages at 210 °C for 72 hours and 400 °C for 48 hours to mix Cu and Sn, followed by a final stage at temperature *θ* for 48 hours to form the Nb_3_Sn phase. We varied *θ* from 599 to 752 °C. The dwell duration at *θ* was kept constant in this study so as to investigate the effects of one HT parameter at a time.

## Results

We used a Cu-Be Walters spring to strain samples *in-situ*^57–59^. Information on the apparatus is provided in the *Methods* section. For each sample (of helical form), we determined *I*_c_ values for three sample locations, each being one full sample turn (or segment) about 8 cm long. Measurements were made while the sample was immersed in liquid helium at a temperature of 4.04 to 4.07 K and subjected to an external magnetic field of 15 T provided by a superconducting solenoid. For each billet, we measured one to three samples (i.e., three to nine segments) per HT. For this study, *I*_c_(*ε*) measurements were made on 94 samples in total. Values of *I*_c_ were determined at the electric field criterion *E*_c_ of 0.1 μV/cm.

Details of the *RRR* apparatus are also given in the *Methods* section. Measurements of sample resistance *R* as a function of sample temperature *T* were made to determine *RRR*, defined as the ratio of *R* at 293 K to that at 18 K. For each billet, we measured three samples per HT. For this study, *RRR* measurements were made on 81 samples in total. Values of *RRR* in the literature are generally quite scattered, so the results we present here may not be representative of those of a large number of billets of a given design.

Expanded uncertainties (*k* = 2) due to random effects in estimating *θ*, *I*_c_, *ε*_irr,0_, and *RRR* were 3 °C, 2%, 0.03% strain, and 30%, respectively. The uncertainties for *I*_c_, *ε*_irr,0_, and *RRR* are based on type A evaluations of uncertainty, whereas the uncertainty for *θ* is based on type B evaluation of uncertainty^60^.

### Effects of heat-treatment temperature on *ε*_irr,0_ and *RRR*

**Figure 1:**
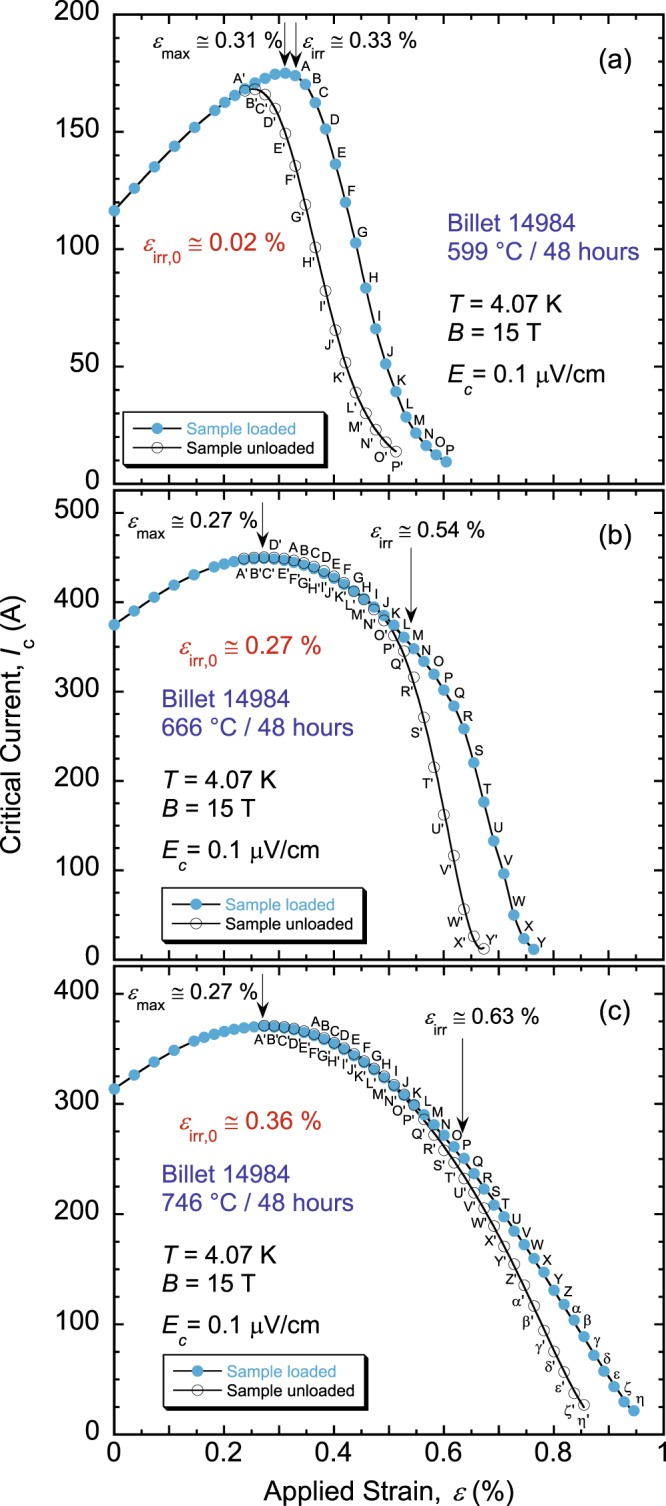
Examples of *I*_c_(*ε*) at 4.07 K and 15 T for samples of a reduced-Sn, Ti-doped, RRP Nb_3_Sn wire 14984, heat-treated for 48 hours at (**a**) 599 °C, (**b**) 666 °C, and (**c**) 746 °C. The intrinsic irreversible strain limit *ε*_irr,0_ (=*ε*_*irr*_ − *ε*_*max*_) has a strong dependence on the heat-treatment temperature *θ*. The sample was loaded and partially unloaded (by constant axial-strain steps of about 0.09%) to obtain the “loaded” and “unloaded” *I*_c_(*ε*) curves, represented by solid and empty symbols, respectively. Corresponding loaded and unloaded points are labelled by unprimed and primed letters, respectively. *ε*_*irr*_ is defined as the applied strain that produces the first splitting of these two curves. *ε*_max_ is the applied strain that compensates for the sample’s pre-compressive strain.

Examples of *I*_c_(*ε*) data are provided in Fig. 1. These particular results are for the reduced-Sn wire 14984; Fig. 1(a,c) are for the *θ* spectrum extremes used for this billet, 599 and 746 °C, respectively, and Fig. 1(b) is for the intermediate value 666 °C. Other *I*_c_(*ε*) examples are published in^46^ for billets 11976-1 and 13711-2. Strain applied to the sample is increased incrementally until *I*_c_ reaches its peak value *I*_c-max_ at strain *ε*_max_ that compensates for the Nb_3_Sn compressive pre-strain. The latter develops during cool-down of the sample from *θ* to 4 K, and originates from the mismatch of thermal expansion coefficient of Nb_3_Sn with respect to those of the other wire components and that of Cu-Be spring material to which the sample is soldered. Around *ε*_max_, we unload strain partially and remeasure *I*_c_ to check its reversibility with strain. This operation is repeated multiple times while increasing strain gradually until *I*_c_ is driven close to zero. From the “loaded” and “unloaded” (solid- and empty-symbol) curves thus obtained, we determine the irreversible strain limit *ε*_irr_ that produces the first splitting of the two curves (see details in the *Methods* section)^42^. The partial-unloading step is kept constant at 0.09% throughout the experiments; it is small enough to minimize *I*_c_ increases upon unloading (for *ε* < *ε*_irr_) due to the three-dimensional strain effects^43,61^, and large enough to reveal *I*_c_(*ε*) irreversibility. Locations of *ε*_max_ and *ε*_irr_ are marked by arrows in Fig. 1(a–c).

Our values of *ε*_max_ and *ε*_irr_ are artificially high because the thick Cu-Be spring dominates the thermal contraction of the assembly (spring + sample) and puts an additional compressive pre-strain on Nb_3_Sn upon cooling to 4 K. These values can also change slightly from sample to sample depending on how tight the sample is made on the spring before soldering. Nevertheless, *I*_c_ versus intrinsic strain *ε*_0_ (=*ε* − *ε*_max_) is not affected by the spring material’s differential thermal contraction or by the sample mounting^35,62^. Therefore, the same should hold for the values of the intrinsic irreversible strain limit *ε*_irr,0_ (=*ε*_irr_ − *ε*_max_). Absolute values of *ε*_max_ (and *ε*_irr_) provided herein should not be taken as a direct measure of strain margins for the wires. In fact, the actual values of *ε*_max_ for RRP wires are rather small due to the high Nb_3_Sn fraction as compared to moderate-*J*_c_ Nb_3_Sn conductors^63^. Thus, *ε*_irr,0_ values are appropriate to gage the strain resilience of the wires as investigated here.

**Figure 2:**
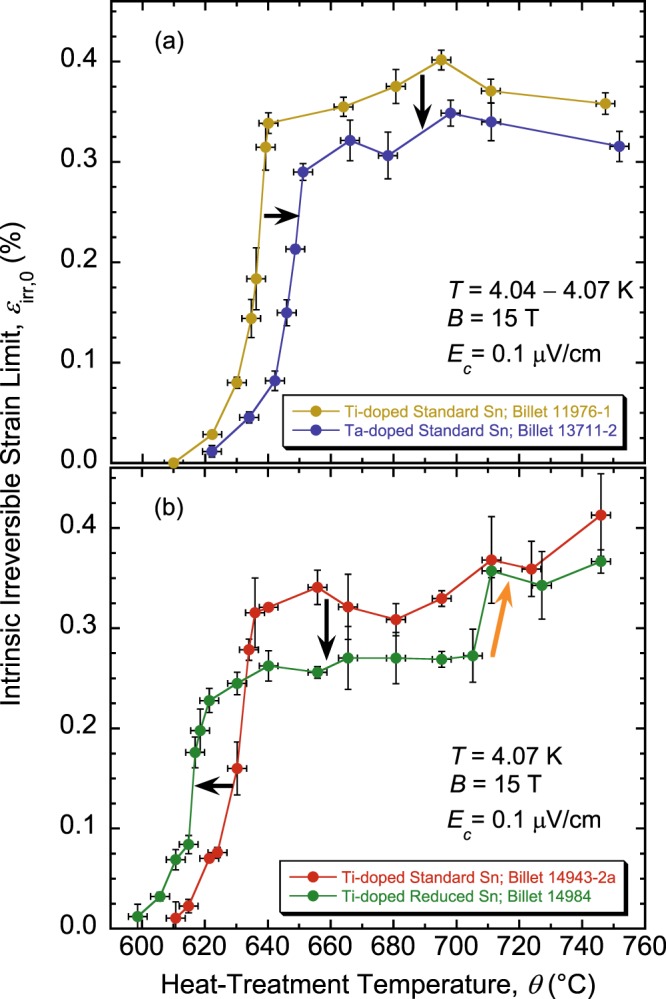
Behavior of *ε*_irr,0_(*θ*) showing the strain irreversibility cliff in four RRP Nb_3_Sn (**a**) standard-Sn, Ti- or Ta-doped, billets 11976-1 and 13711-2, respectively, and (**b**) standard- or reduced-Sn, Ti-doped, billets 14943-2a and 14984, respectively. The arrows in black indicate changes in the SIC location and height with the doping element and amount of Sn. The arrow in orange highlights the second precipitous step in the double-cliff structure of SIC for billet 14984, as explained in the text. The error bars shown for *ε*_irr,0_ values correspond to twice the standard error of their respective mean, $2s/\surd n$, where *s* is the standard deviation and *n* is the number of segments used to calculate the mean. The error bars for *θ* values correspond to ±3 °C.

Values of *ε*_irr,0_ are displayed in Fig. 1(a–c) in red for their corresponding billet and HTs. The complete dependence of *ε*_irr,0_ on *θ* is depicted in Fig. 2(a) for the standard-Sn, Ti- or Ta-doped wires, and in Fig. 2(b) for the standard- or reduced-Sn, Ti-doped wires. Each point represents an average value of *ε*_irr,0_ over one to three samples (i.e., three to nine segments) per HT. For all four wires, *ε*_irr,0_ exhibits a precipitous and large change with *θ*, indicating a transition of Nb_3_Sn from a highly brittle state, where irreversible effects start as soon as Nb_3_Sn is subjected to a tensile strain of any measurable amount, to a more strain-resilient state where changes of *I*_c_(*ε*) remain reversible up to a tensile strain close to 0.4%. This behavior is the *strain irreversibility cliff* (SIC)^46^; it is confirmed here for the four RRP Nb_3_Sn billets. These results clearly show that *θ* (or HT schedule in general) has a major influence on *ε*_irr,0_.

For the four billets, values of *ε*_irr,0_ on the SIC plateau are above the required value of 0.25%. The cliff extends over a narrow temperature range of ≈23 to 29 °C, and its bulk part (excluding the tail) is much steeper as it occurs over only 10 to 15 °C depending on the billet (see Fig. 2). The shift of SIC between the two standard-Sn, Ti-doped billets 11976-1 (Fig. 2(a)) and 14943–2a (Fig. 2(b)) is about 5 °C, indicating that the temperature location of SIC is fairly reproducible for fairly similar wires. In comparison to standard-Sn, Ti-doped wires, SIC is shifted to higher temperatures by 10 to 12 °C for the Ta-doped wire (Fig. 2(a)) and is shifted to lower temperatures by 11 to 14 °C for the reduced-Sn wire (Fig. 2(b)) [see horizontal black arrows in Fig. 2(a,b)]. Also, the height of the cliff is lower for the standard-Sn, Ta-doped, or reduced-Sn, Ti-doped wires [see vertical black arrows in Fig. 2(a,b)]. Nevertheless, for the reduced-Sn wire, *ε*_irr,0_ improved further and precipitously when *θ* is increased beyond 705 °C [see orange arrow in Fig. 2(b)], revealing a double-cliff structure for this particular billet, though the second step is much smaller than the main cliff height. We do not know yet if this double-cliff structure is typical of reduced-Sn billets. These differences from billet to billet may provide clues regarding the origins of SIC. This paper is principally focused on treating the implications of SIC on applications.

**Figure 3:**
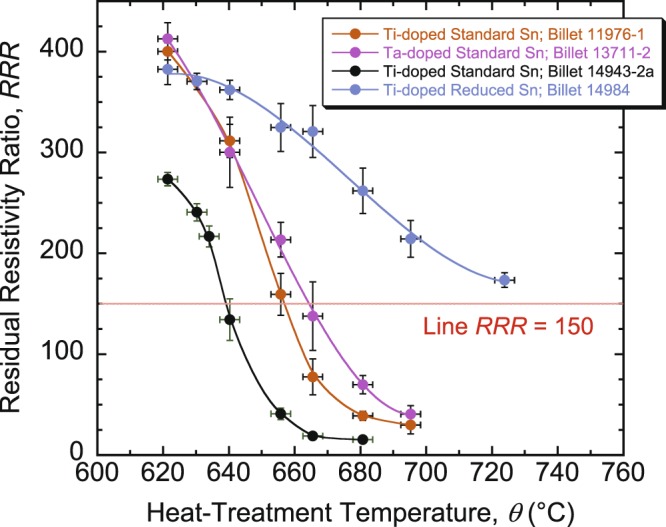
Results of *RRR* as a function of *θ* for four RRP Nb_3_Sn standard- or reduced-Sn, Ti- or Ta-doped wires. *RRR* decreases strongly with *θ*, especially for the standard-Sn billets. The red line delimits *RRR* = 150, the minimum value required for the LHC magnets. It is crossed at *θ* ≈ 639, 656, 664, and (approximately projected) 745 °C for billets 14943-2a, 11976-1, 13711-2, and 14984, respectively. The error bars shown for *RRR* values correspond to twice the standard error of their respective mean, $2s/\surd n$, where *s* is the standard deviation and *n* is the number of samples used to calculate the mean. The error bars for *θ* values correspond to ±3 °C. Note that the results presented here may not be representative of those of a large number of billets of a particular design, given the usually large scatter in *RRR* values.

The results of *RRR*(*θ*) are depicted in Fig. 3, where each data point represents an average value of *RRR* over three samples. Figure 3 illustrates the strong decline of *RRR* as *θ* is increased, especially for the standard-Sn billets. The red line delimits *RRR* = 150, and is crossed at *θ* ≈ 639, 656, 664, and (approximately projected) 745 °C for billets 14943-2a, 11976-1, 13711-2, and 14984, respectively. (This temperature is noted below as *θ*_*RRR*_). The reduced-Sn billet shows a significantly higher *RRR* that stays above 150 for an extended range of *θ* as compared to the standard-Sn billets.

### Electro-mechanical stability (EMS) criterion

As discussed in^46^, the narrowness of the *θ* range where SIC occurs may have challenging implications for the heat-treatment of large magnets. Reducing *θ* to promote the strand’s magneto-electrical stability will be at the expense of the strand’s strain properties if *ε*_irr,0_ is not positioned away from the SIC tip somewhere on the SIC main plateau. The magneto-electrical (or electrical for simplicity) and mechanical requirements are in conflict, and we need to find HT conditions that achieve them both. In our analysis, we build on the requirements *RRR* ≥ 150 and *ε*_irr,0_ ≥ 0.25%, but, for the latter, we put more emphasis on staying away from the cliff to ensure that the inevitable temperature gradient across the magnet during heat-treatment and furnace-temperature imprecisions do not produce weak *ε*_irr,0_ anywhere in the magnet. This analysis is flexible enough to accommodate other applications if *RRR* requirements are different.

**Figure 4:**
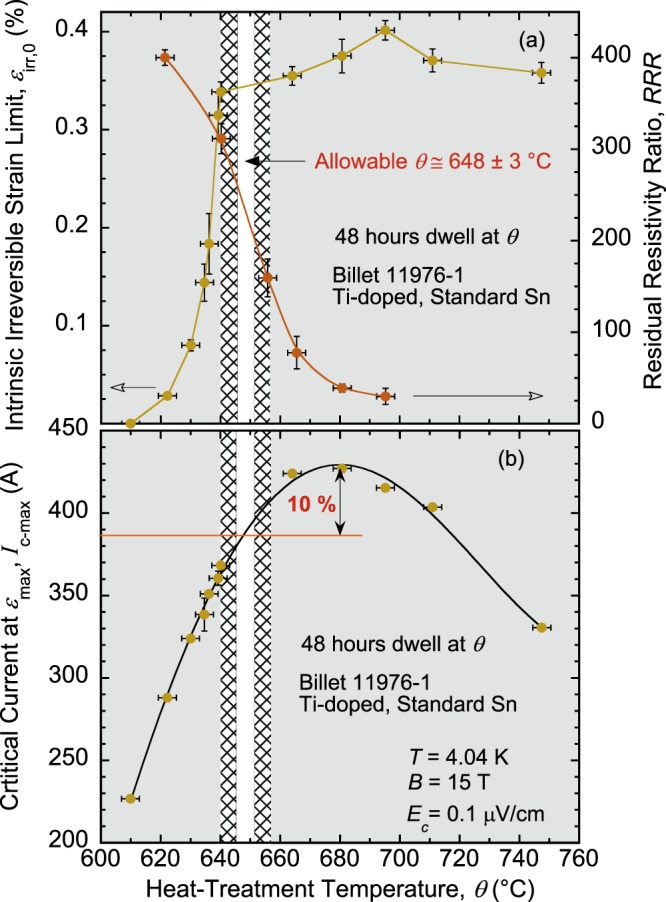
(**a**) Comparisons of *ε*_irr,0_(*θ*) and *RRR*(*θ*) dependences for the RRP Nb_3_Sn standard-Sn, Ti-doped, billet 11976-1. The arrows near the bottom point to the Y-axis corresponding to each curve. Areas shaded in gray on the left and on the right delimit the domains where *θ* < *θ*_Cliff_ (unsatisfactory mechanical properties), and *θ* > *θ*_*RRR*_ (unsatisfactory electrical properties), respectively. The hatched areas in the remaining window represent temperature safety margins of a width *δθ* ≈ 5 °C on each side. The residual domain between the hatched areas defines the allowable temperature window for an optimal HT, as defined in Eq. (2) of the EMS criterion. For billet 11976-1, the allowable *θ* is 648 ± 3 °C. (**b**) Restrictions of the EMS criterion on *θ* mean that this wire will be used at approximately 10% below its full *I*_c_ potential. The error bars shown for *ε*_irr,0_, *RRR*, and *I*_c-max_ values correspond to twice the standard error of their respective mean, $2s/\surd n$, where *s* is the standard deviation and *n* is the number of segments used to calculate the mean. The error bars for *θ* values correspond to ±3 °C.

**Figure 5:**
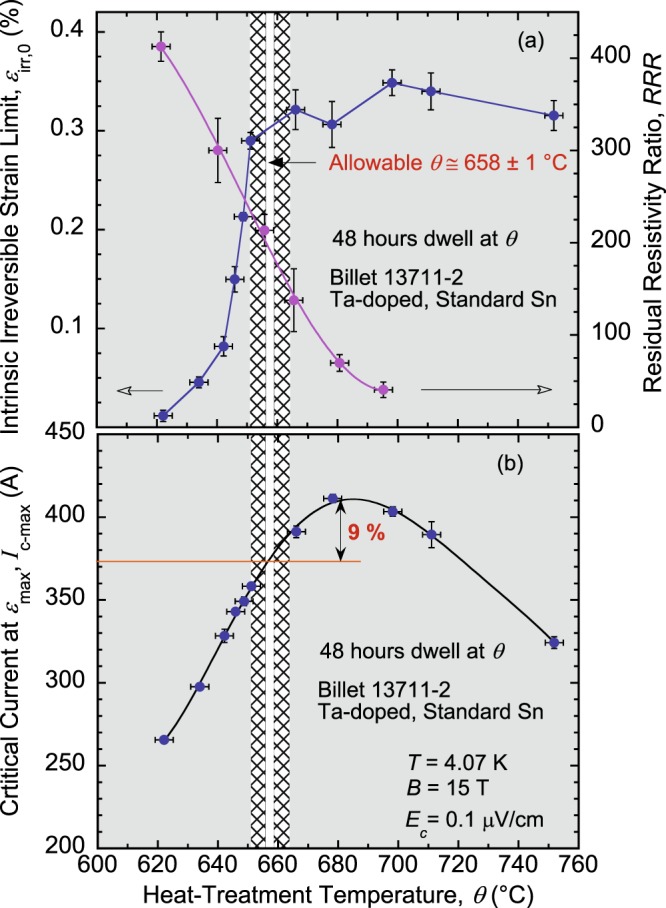
(**a**) Comparisons of *ε*_irr,0_(*θ*) and *RRR*(*θ*) dependences for the RRP Nb_3_Sn standard-Sn, Ta-doped, billet 13711-2, presented as in Fig. 4. The arrows near the bottom point to the Y-axis corresponding to each curve. For billet 13711-2, the allowable *θ* is 658 ± 1 °C. (**b**) Restrictions of the EMS criterion on *θ* mean that this wire will be used at approximately 9% below its full *I*_c_ potential. The error bars shown for *ε*_irr,0_, *RRR*, and *I*_c-max_ values correspond to twice the standard error of their respective mean, $2s/\surd n$, where *s* is the standard deviation and *n* is the number of segments used to calculate the mean. The error bars for *θ* values correspond to ±3 °C.

**Figure 6:**
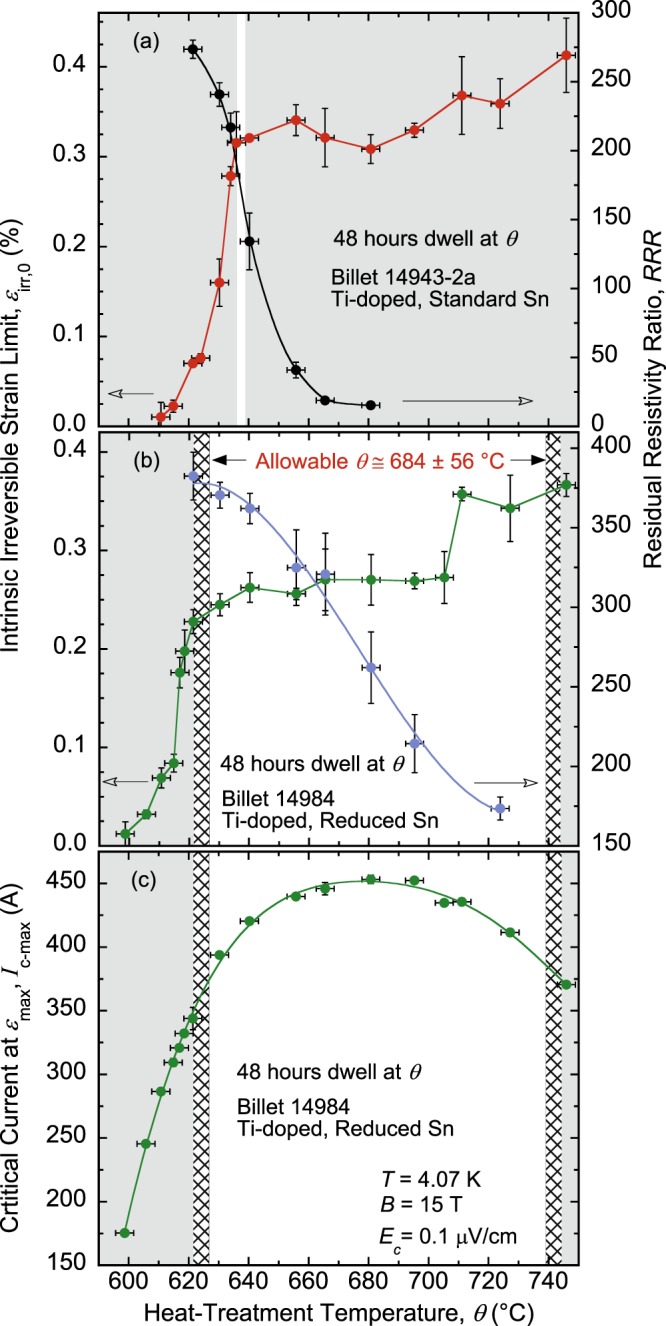
Comparisons of *ε*_irr,0_(*θ*) and *RRR*(*θ*) dependences for the RRP Nb_3_Sn (**a**) standard-Sn, Ti-doped, billet 14943-2a. and (**b**) reduced-Sn, Ti-doped, billet 14984, presented as in Fig. 4. The arrows near the bottom of (**a**) and (**b**) point to the Y-axis corresponding to each curve. The EMS criterion is not met for billet 14943-2a and shows its limited suitability for such HTs. In contrast, the allowable *θ* of 684 ± 56 °C is very wide for billet 14984. (**c**) The allowable *θ* for billet 14984 is centered very close to the optimum value of *θ* (≈ 680 °C) where *I*_c_ is maximal. No trade-off is needed between *I*_c_ and *RRR*. The error bars shown for *ε*_irr,0_, *RRR*, and *I*_c-max_ values correspond to twice the standard error of their respective mean, $2s/\surd n$, where *s* is the standard deviation and *n* is the number of segments used to calculate the mean. The error bars for *θ* values correspond to ±3 °C.

To link *RRR*(*θ*) and *ε*_irr,0_(*θ*) results for analysis, we define *θ*_*RRR*_ as the temperature where *RRR* crosses the required minimum value (150 in the case of LHC magnets) and *θ*_Cliff_ as the temperature where the precipitous drop of *ε*_irr,0_ starts to occur (tip or onset of SIC). The choice of *θ* must be such that the wire fulfills both requirements for electrical stability (*RRR* ≥ 150 for LHC magnets; it could be a different value for another application) and mechanical stability (*ε*_irr,0_ at the top of SIC). First, billet properties must be such that *θ*_Cliff_ is lower than *θ*_*RRR*_ with enough margin; then, the operator must choose *θ* between these two values. We capture these recommendations in what we call the *electro-mechanical stability (EMS) criterion* as:

1 $${\theta }_{{\rm{Cliff}}}+\delta \theta  < {\theta }_{RRR}-\delta \theta ,$$

where *δθ* is introduced to account for uncertainties in *θ* arising from possible inhomogeneity and inaccuracy of furnace temperature. If a wire (considered for fabricating a magnet) meets this requirement, then *θ* must be chosen such that

2 $${\theta }_{{\rm{Cliff}}}+\delta \theta  < \theta  < {\theta }_{RRR}-\delta \theta ,$$

thus setting the allowable window for best HT. We define the EMS criterion by both equations (1) and (2), even though fulfilling Eq. (2) implies the same for Eq. (1). We wanted to indicate that some billets may be intrinsically limited to fulfil Eq. (1), in which case fabricating a magnet from such billet should not be even considered (let alone heat-treating it). We believe the EMS criterion is a valuable tool to inspect billets *before* considering their use in magnets. We illustrate this concept in Figs 4–6 for the four wires, for the LHC magnets.

In Fig. 4(a), *ε*_irr,0_(*θ*) and *RRR*(*θ*) are plotted together for the standard-Sn, Ti-doped billet 11976-1 (the arrows near the bottom point to the Y-axis corresponding to each curve). The areas shaded in gray delimit the domains where *θ* < *θ*_Cliff_ (unsatisfactory mechanical properties) and *θ* > *θ*_*RRR*_ (unsatisfactory electrical properties). The hatched areas in the remaining window (in Fig. 4a) represent temperature safety margins of a width *δθ* ≈ 5 °C on each side to ensure that conductor performance is sufficiently away from the SIC tip and away from getting below *RRR* of 150. Thus, the residual domain between the hatched areas (pointed to by an arrow in Fig. 4(a)) defines the allowable temperature window for an optimal HT, as defined in Eq. (2). In this case of billet 11976-1, the allowable *θ* is 648 ± 3 °C; a very narrow range that might be difficult to apply for heat treating large magnets (4 to 8 meters long in the case of HL-LHC). The value of 5 °C for *δθ* is arbitrary and used here for illustration purposes. Actual value of *δθ* can be adjusted depending on the quality of the furnace used and on the size of the magnet. It can be also adjusted to reflect *θ*_Cliff_ and *θ*_*RRR*_ variations among a large number of billets of a given design (when such statistical data are available).

In Fig. 4(b), the dependence of *I*_c-max_ on *θ* is shown for same billet 11976-1 (see ref.^46^ for more details on this dependence and on the choice of *I*_c_ at this particular strain reference *ε*_max_). The gray and hatched areas of Fig. 4(a) are pasted in Fig. 4(b) to evaluate the consequences on *I*_c_ of the EMS criterion’s HT restrictions. It appears that staying within the allowable HT window means that this wire will be used at approximately 10% below its full *I*_c_ potential. Figure 4(b) reveals the trade-off between the electro-mechanical and transport properties for this particular billet, for a HT conducted at the optimal *θ* (=648 °C) for a duration of 48 hours. We will comment on the effect of HT duration later.

Analysis for the standard-Sn, Ta-doped billet 13711-2 is depicted in the same way in Fig. 5. For this billet, the allowable HT window is 658 ± 1 °C (Fig. 5(a)). It is located at a temperature 10 °C higher than for billet 11976-1 but is narrower, making it even more difficult to apply such HT. As shown in Fig. 5(b), this wire will be at approximately 9% below its full *I*_c_ potential. The case of the standard-Sn, Ti-doped billet 14943-2a illustrated in Fig. 6(a) is the worst. The HT window is too small, even smaller than the safety margin *δθ*. Hence, the EMS criterion is not met and, as such, there is no temperature window to secure an optimal HT (at 48-hour dwell time) that will satisfy both the electrical and mechanical requirements. Therefore, we suggest that such a billet be discarded to avoid fabricating magnets with poor properties and save valuable funds. This particular billet may not be representative of the standard-Sn billets produced, considering its low values of *RRR* (see Fig. 3), but is illustrative of the implications of SIC that could possibly happen. Furthermore, existence of outlier billets in a large production volume cannot be excluded.

The narrowness of the allowable HT window for the three standard-Sn billets, dictated by the EMS criterion, is not desirable. It might be a general trend for standard-Sn billets, but we cannot be certain as there is some *RRR* variability from billet to billet. Nevertheless, alternative HTs with lower temperatures for longer dwell time might render such billets more suitable. By lowering *θ* below *θ*_Cliff_, *ε*_irr,0_ will decrease but perhaps will be restored if the dwell time is increased sufficiently. However, we should also consider the dependence of *RRR* on dwell time. Lowering *θ* will increase *RRR*, but increasing the dwell time will tend to decrease *RRR*. So, it is a matter of finding the right balance between *θ* and dwell time to achieve high *ε*_irr,0_ and high *RRR*. This deserves a study of its own to supplement the results presented here. Effects on the allowable HT window of a simplified HT scheme that has two stages (instead of three) to control the Sn-Nb-Cu Nausite phase are also worthy of a systematic study^19^.

For the reduced-Sn billet, in contrast, the situation is far better. As depicted in Fig. 6(b), the allowable *θ* is 684 ± 56 °C—a very wide range. Moreover, it is centered very close to the optimum value of *θ* (≈ 680 °C) where *I*_c_ is maximal (see Fig. 6(c)). Therefore, no trade-off is needed between *I*_c_ and *RRR* for such billet except for the lowering of *I*_c_ due to the reduced content of Sn (see the discussion below). Such a billet seems very suitable. It is fortunate that HL-LHC will use the reduced-Sn RRP billets design.

**Figure 7:**
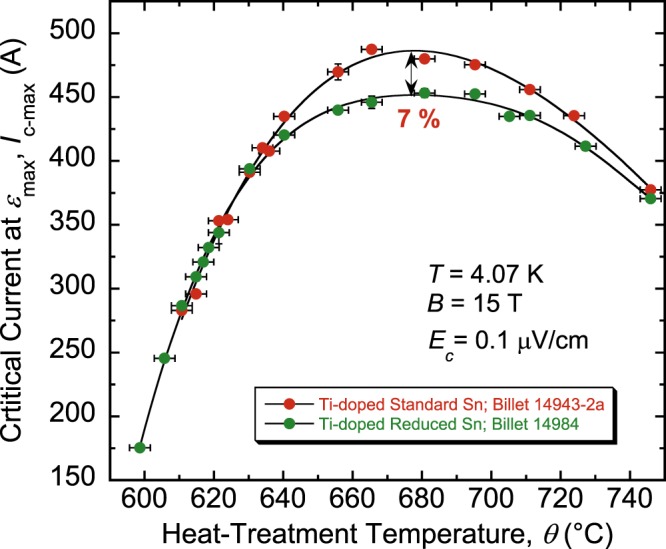
Dependence of *I*_c-max_(*θ*) for the RRP Nb_3_Sn standard- or reduced-Sn, Ti-doped, 14943-2a and 14984 wires, respectively. Values of *I*_c-max_ are the same for both billets for *θ* ≤ 630 °C. Differences appear above 630 °C and culminate at about 7% around 680 °C (where *I*_c-max_ is maximal for both billets) in favor of the standard-Sn billet. The non-Cu areas for both wires is the same, 46.5% of the wires cross-sections. The error bars shown for *I*_c-max_ values correspond to twice the standard error of their respective mean, $2s/\surd n$, where *s* is the standard deviation and *n* is the number of segments used to calculate the mean. The error bars for *θ* values correspond to ±3 °C.

Comparison of *I*_c-max_ between the standard- and reduced-Sn billets 14943-2a and 14984 is displayed as a function of *θ* in Fig. 7. These two billets are well suited for a direct comparison of *I*_c_ because they have the same non-Cu area (Table 1). Values of *I*_c-max_ are the same for both billets for *θ* ≤ 630 °C. Differences appear above 630 °C and culminate at about 7% around 680 °C in favor of the standard-Sn billet. However, considering that the reduced-Sn billet can be used with no *I*_c_ trade-off and that the standard-Sn billet may be used but at less than its full *I*_c_ potential due to the EMS-criterion restrictions, the reduced-Sn billet is in fact the better option even in terms of *I*_c_. If higher *I*_c_ is needed, we could envision a billet design having an amount of Sn reduced by less than 6% compared to the standard-Sn design (for example 4% or so). Such design may increase *I*_c_ but may also shrink the allowable HT window in comparison to that of billet 14984 (Fig. 6(b)), though we speculate that it may still be wide enough. Also, it might be possible that such a modified billet will have a higher *ε*_irr,0_ than billet 14984, somewhere between the values for billets 14984 and 14943-2a (Fig. 2(b)).

The US HL-LHC AUP in collaboration with CERN decided that HT of magnets be made at 665 °C (for a dwell time <50 hours) to stay away from SIC^23^. Whereas this choice is consistent with what we presented here so far, below we show that an increase of this temperature by about 15 to 30 °C may have some additional benefits.

### Searching for useful information beyond the irreversible strain limit

The reversible effects of strain have been studied extensively since the 1970s, once the high sensitivity of Nb_3_Sn properties to axial strain became evident^64^. Comparatively, the strain boundary of these reversible effects, *ε*_irr,0_ (or *ε*_irr_), has received very little attention. As for the irreversible effects themselves, they have probably been considered not worth studying beyond using the data to determine *ε*_irr,0_ (or *ε*_irr_). This limit is generally considered as the intrinsic strain where cracks start to form in Nb_3_Sn. Hence, disregarding data beyond it is understandable, considering that applications should not be designed to exceed this limit^41^.

In a recent report, we showed some evidence that *I*_c_ degradation rate in the irreversible regime also depends on HT conditions^46^. This is the reason why we conducted measurements of *I*_c_(*ε*) far beyond *ε*_irr_ for the samples presented here, until *I*_c_ was driven close to zero as in Fig. 1. In Fig. 1(b), for example, the shape of *I*_c_(*ε*) (loaded curve) for 666 °C has an “elbow” marking a severe degradation of *I*_c_ that does not start immediately at *ε*_irr_. This feature in *I*_c_(*ε*) dependence is also present for several HTs below 666 °C, and more pronounced, but disappears for HTs above 666 °C as in Fig. 1(c) for 746 °C.

**Figure 8:**
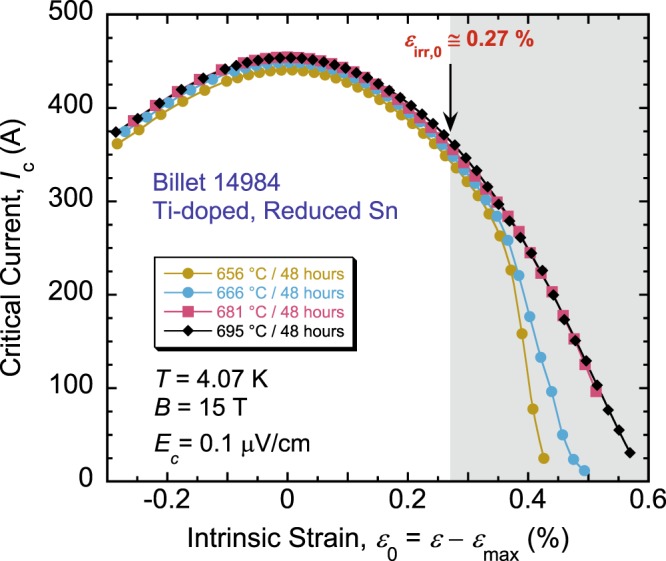
Comparisons at 4.07 K and 15 T of *I*_c_(*ε*_0_) loaded curves for samples of the RRP reduced-Sn, Ti-doped, Nb_3_Sn wire 14984 heat-treated for 48 hours at 656, 666, 681, and 695 °C, for the specific measurements protocol described in the text. Samples reacted at 666–695 °C have practically the same *I*_c_ values for any given intrinsic strain *ε*_0_, even beyond *ε*_irr,0_, until the sample reacted at 666 °C starts showing a severe drop of *I*_c_. This drop is more pronounced for the sample reacted at 656 °C, indicating its gradual evolution with lowering temperature *θ*. Values of *ε*_irr,0_ are almost the same for the four samples. The area shaded in gray on the right corresponds to the irreversible strain regime (*ε*_0_ > *ε*_irr,0_). These results indicate that it may be beneficial to heat-treat magnets for HL-LHC at a temperature between 680 and 695 °C (for a dwell time of 48 hours), instead of the planned 665 °C, to contain the strain-induced *I*_c_ degradation beyond *ε*_irr,0_.

In Fig. 8, we show a comparison of *I*_c_(*ε*_0_) for HTs done at 656, 666, 681, and 695 °C, for the specific measurements protocol (described above) that we applied in all these experiments. Note that the curves represent raw *I*_c_ data (not normalized), plotted as a function of the intrinsic strain (*ε*_0_ = *ε* − *ε*_max_) to eliminate slight differences of *ε*_max_ from sample to sample. The values and behavior of *I*_c_(*ε*_0_) are very similar for the three samples reacted at 666–695 °C, even past *ε*_irr,0_, until the elbow feature appears for the sample reacted at 666 °C that distinguishes it from the other two samples. This feature is even more pronounced for the sample reacted at 656 °C, indicating its progressive evolution with lowering temperature *θ*. The four samples have about the same value of *ε*_irr,0_ (Fig. 2(b)).

Disappearance of the severe drop of *I*_c_(*ε*_0_) for samples reacted at 681 °C and above may possibly indicate an increase of the fracture toughness of Nb_3_Sn that perhaps becomes more resistant to additional cracking and crack propagation (at a given strain) in these samples. Fracture toughness measurements on individual filaments are needed to verify this assumption. These observations suggest that it may be judicious to increase *θ* for the LHC magnets from 665 °C to somewhere between 680 and 695 °C (when the dwell time at *θ* is 48 hours) to contain *I*_c_ irreversible degradation, especially that this increase in *θ* keeps it still within the allowable HT window (Fig. 6(b)). Even though the design of these magnets should be such that *ε*_irr,0_ is not exceeded, this adjustment of *θ* would provide an extra precaution.

## Conclusion

We coupled studies of *I*_c_(*ε*) and those of *RRR* for various RRP Nb_3_Sn billets heat-treated at different temperatures from 599 to 752 °C for 48 hours. The billets were either standard- or reduced-Sn, doped with either Ti or Ta, of the type intended for use in particle-accelerator magnets for HL-LHC. They all exhibited an abrupt change of *ε*_irr,0_ as a function of *θ*, known as the strain irreversibility cliff. The approach of combining the two studies allowed us to comprehensively assess the implications of SIC on restricting the HT conditions. We introduced the electro-mechanical stability criterion that takes into account both requirements for electrical (*RRR* ≥ 150) and mechanical (*ε*_irr,0_ away from SIC) stability for best outcome. For the standard-Sn billets, fulfilling these conflicting requirements yields a significant narrowing of the allowable HT temperature window that is impractical. On the other hand, the reduced-Sn billets offer a significantly wider choices for HT, not only for ensuring that *RRR* ≥ 150 and *ε*_irr,0_ is located at the top of SIC, but also for containing the strain-induced irreversible degradation of *I*_c_ beyond *ε*_irr,0_. This study suggests that HT of magnets for HL-LHC, made of reduced-Sn RRP billets having a Nb/Sn ratio of 3.6 and 108/127 restacking architecture, be conducted at a temperature of 680 to 695 °C, about 15 to 30 °C higher than the 665 °C (for a dwell time <50 hours) that is targeted by HL-LHC project.

Finally, we highly recommend investigating whether the effects of *transverse* compression are also dependent on HT conditions. This will allow the magnet designer to account for effects of HT on conductor resilience against the various stress components in a magnet. Of course, the *reversible* effects should be taken into account as well, and be measured or projected accurately through *I*_c_ parametrization within the reversible strain regime (*ε*_0_ ≤ *ε*_irr,0_) as a function of magnet operating conditions of strain, temperature, and magnetic field^37^. A spreadsheet was created to facilitate these calculations^65^.

## Methods

### Apparatus for *I*_c_(*ε*) measurements

**Figure 9:**
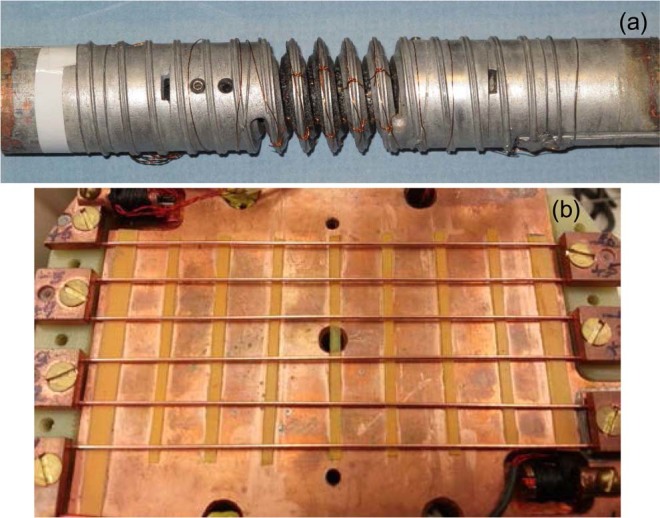
(**a**) A Walters spring made of Cu-Be onto which a Nb_3_Sn sample is soldered. This device is used to apply strain to the sample by twisting one end of the spring with respect to the other end. The spring diameter is 25 mm. It has four turns, cut out in a T-section shape designed to maximize the elastic strain range of the spring to ±1%. Current splices are visible at each end of the spring, where the sample turns are doubled up. These splices were cut out from spare turns of the sample. (**b**) Device for sample resistance measurements to determine *RRR*. It accommodates six straight samples mounted in series. Each sample is about 13 cm long.

Transport measurements of *I*_c_ as a function of applied axial strain *ε* were conducted by use of a Walters spring made of Cu-Be (see Fig. 9(a))^57–59^. This spring device had four turns with a T-section profile, designed such that the spring behaves elastically over a wide strain range from −1% to +1%^58,59^. By twisting one spring end with respect to the other end, the spring’s outer surface where the sample was attached can be subjected to either a tensile or a compressive axial strain^57–59^. The sample was first mounted on a stainless-steel mandrel for heat treatment, then transferred onto the spring carefully and soldered to it along its full length at ≈200 °C. The sample length prepared (~2.5 m) allowed for multiple spare turns that we cut and used as current splices (visible in Fig. 9(a)). They were soldered to the sample ends to facilitate current transfer into the sample with minimal contact resistance. Multiple pairs of instrumentation leads were attached to the sample to monitor voltage *V* during ramping of electric current *I* through the sample. Each of the three main pairs covered one of the sample turns (≈8 cm long each) that were on the spring turns. Values of *I*_c_ were determined from *V*-*I* characteristics at various electric-field criteria *E*_c_. Data shown here are for 0.1 μV/cm that is a more commonly used criterion.

To extract the values of *ε*_max_ and *ε*_irr_, a polynomial function (at varying orders) was fitted to the loaded *I*_c_(*ε*) data excluding significantly degraded data points (i.e. strain points that yielded a degradation in excess of 5% or so upon strain partial-unloading). The roots of this polynomial function were then found through derivation, and the realistic root value was identified as that of *ε*_max_. The high density of acquired data points around *ε*_max_ (see Fig. 1) made identification of the realistic root very simple. For *ε*_irr_, the residuals (*I*_c-measured_/*I*_c-predicted_ − 1) for the unloaded points—*I*_c-predicted_ being *I*_c_ calculated by use of the polynomial fitting function assuming no *I*_c_ irreversible degradation—were determined as a function of *ε*. Points with residuals around 1% were then extrapolated to find the strain value with a residual of 0. This strain, at the onset of *I*_c_ irreversible degradation, was identified as *ε*_irr_. It corresponds to strain that caused the first splitting of the loaded and unloaded curves (see Fig. 1). More details are provided in^42^.

### Apparatus for *RRR* measurements

Samples for resistivity measurements were mounted on the apparatus shown in Fig. 9(b). This *RRR* apparatus was designed to accommodate six straight samples mounted in series. Pressure contacts were used to connect the current and voltage contacts to each sample. Voltage-tap pairs on each sample were separated by 10 cm. The probe was inserted into a cold cryostat and lowered very slowly to gradually cool the probe with the ambient helium gas. The samples and thermometers were positioned horizontally to minimize temperature gradients. In addition, and for the same purpose, samples were enclosed between two nesting copper plates (only one Cu plate is shown in Fig. 9(b)). An excitation current of about 0.1 A was run through the samples and voltage across each of them was measured to determine their respective resistance. Sample resistance *R* was measured as a function of sample temperature *T*, from room temperature *T*_0_ down to temperatures low enough for the sample to become superconducting. *RRR* was defined as the ratio of *R* at 293 K to that at 18 K (just before the superconducting transition). The value of *R*(293 K) was extrapolated from *R*(*T*_0_) by use of the empirical equation^66,67^:

3 $$R(293\,K)=\frac{R({T}_{0})}{[1+0.00393\times ({T}_{0}-293)]}$$

For HT (prior to measurements), samples were inserted individually into small-diameter alumina tubes to keep them straight.

## References

[1] Ballarino A, Bottura L (2015). Targets for R&D on Nb_3_Sn conductor for high energy physics. IEEE Trans. Appl. Supercond..

[2] Cooley LD, Ghosh AK, Dietderich DR, Pong I (2017). Conductor specification and validation of high-luminosity LHC quadrupole magnets. IEEE Trans. Appl. Supercond..

[3] Rossi L (2010). Superconductivity: its role, its success and its setbacks in the Large Hadron Collider of CERN. Supercond. Sci. Technol..

[4] Bottura L, Godeke A (2012). Superconducting materials and conductors: fabrication and limiting parameters. Rev. Accl. Sci. Technol..

[5] Gourlay SA (2018). Superconducting accelerator magnet technology in the 21^st^ century: A new paradigm on the horizon?. Nucl. Instrum. Meth. Phys. Res. A.

[6] Parrell JA, Zhang Y, Field MB, Cisek P, Hong S (2003). High field Nb_3_Sn conductor development at Oxford Superconducting Technology. IEEE Trans. Appl. Supercond..

[7] Parrell JA (2009). Internal tin Nb_3_Sn conductors engineered for fusion and particle accelerator applications. IEEE Trans. Appl. Supercond..

[8] Field MB, Zhang Y, Miao H, Gerace M, Parrell A (2014). Optimizing Nb_3_Sn conductors for high field applications. IEEE Trans. Appl. Supercond..

[9] Tarantini C, Lee PJ, Craig N, Ghosh A, Larbalestier DC (2014). Examination of the trade-off between intrinsic and extrinsic properties in the optimization of a modern internal tin Nb_3_Sn conductor. Supercond. Sci. Technol..

[10] Zlobin AV (2005). R&D of Nb_3_Sn accelerator magnets at Fermilab. IEEE Trans. Appl. Supercond..

[11] Dietderich DR (2005). Correlation between strand stability and magnet performance. IEEE Trans. Appl. Supercond..

[12] Ghosh AK, Cooley LD, Moodenbaugh AR (2005). Investigation of instability in high *J*_c_ Nb_3_Sn strands. IEEE Trans. Appl. Supercond..

[13] Barzi E (2005). Instabilities in transport current measurements of Nb_3_Sn strands. IEEE Trans. Appl. Supercond..

[14] Bordini B, Rossi L (2009). Self field instability in high-*J*_c_ Nb_3_Sn strands with high copper residual resistivity ratio. IEEE Trans. Appl. Supercond..

[15] Takala E (2012). An experimental setup to measure the minimum trigger energy for magnetothermal instability in Nb_3_Sn strands. IEEE Trans. Appl. Supercond..

[16] Bordini B, Bottura L, Oberli L, Rossi L, Takala E (2012). Impact of the residual resistivity ratio on the stability of Nb_3_Sn magnets. IEEE Trans. Appl. Supercond..

[17] Ghosh AK (2013). Effect of copper resistivity and filament size on the self-field instability of high-*J*_c_ Nb_3_Sn strands. IEEE Trans. Appl. Supercond..

[18] Bordini B (2013). Magnetization measurements of high-*J*_c_ Nb_3_Sn strands. IEEE Trans. Appl. Supercond..

[19] Sanabria C (2018). Controlling Cu–Sn mixing so as to enable higher critical current densities in RRP^®^ Nb_3_Sn wires. Supercond. Sci. Technol..

[20] Simon, N. J., Drexler, E. S. & Reed, R. P. Properties of copper and copper alloys at cryogenic temperatures. *NIST Monograph* **177** (1992).

[21] Ambrosio G (2013). Test results and analysis of LQS03 third long Nb_3_Sn quadrupole by LARP. IEEE Trans. Appl. Supercond..

[22] Godeke A (2013). A review of conductor performance for the LARP high-gradient quadrupole magnets. Supercond. Sci. Technol..

[23] Ambrosio, G. *et al*. *Design criteria for MQXFA superconducting elements*. US HL-LHC Accelerator Upgrade Project. Document 885-v5. http://us-hilumi-docdb.fnal.gov/cgi-bin/ShowDocument?docid=885.

[24] Ekin JW (1976). Effect of stress on the critical current of Nb_3_Sn multifilamentary composite wire. Appl. Phys. Lett..

[25] Ekin JW (1980). Strain scaling law for flux pinning in practical superconductors. Part 1: Basic relationship and application to Nb_3_Sn conductors. Cryogenics.

[26] ten Haken B, Godeke A, ten Kate HHJ (1999). The strain dependence of the critical properties of Nb_3_Sn conductors. J. Appl. Phys..

[27] Cheggour N, Hampshire DP (1999). Unifying the strain and temperature scaling laws for the pinning force density in superconducting niobium-tin multifilamentary wires. J. Appl. Phys..

[28] Cheggour N, Hampshire DP (2002). The unified strain and temperature scaling law for the pinning force density of bronze-route Nb_3_Sn wires in high magnetic fields. Cryogenics.

[29] Taylor DMJ, Hampshire DP (2005). The scaling law for the strain dependence of the critical current density in Nb_3_Sn superconducting wires. Supercond. Sci. Technol..

[30] Flükiger R, Uglietti D, Abächerli V, Seeber B (2005). Asymmetric behavior of *J*_c_(*ε*) in Nb_3_Sn wires and correlation with the stress induced elastic tetragonal distortion. Supercond. Sci. Technol..

[31] Oh S, Kim K (2006). A scaling law for the critical current of Nb_3_Sn strands based on strong-coupling theory of superconductivity. J. Appl. Phys..

[32] Godeke A, ten Haken B, ten Kate HHJ, Larbalestier DC (2006). A general scaling relation for the critical current density in Nb_3_Sn. Supercond. Sci. Technol..

[33] Ilyin Y, Nijhuis A, Krooshoop E (2007). Scaling law for the strain dependence of the critical current in an advanced ITER Nb_3_Sn strand. Supercond. Sci. Technol..

[34] Markiewicz WD (2008). Comparison of strain scaling functions for the strain dependence of composite Nb_3_Sn superconductors. Supercond. Sci. Technol..

[35] Cheggour N (2012). Strain and magnetic-field characterization of a bronze-route Nb_3_Sn ITER wire: benchmarking of strain measurement facilities at NIST and University of Twente. IEEE Trans. Appl. Supercond..

[36] Bordini B, Alknes P, Bottura L, Rossi L, Valentinis D (2013). An exponential scaling law for the strain dependence of the Nb_3_Sn critical current density. Supercond. Sci. Technol..

[37] Ekin JW, Cheggour N, Goodrich L, Splett J (2017). Unified scaling law for flux pinning in practical superconductors: III. Minimum datasets, core parameters, and application of the extrapolative scaling expression. Supercond. Sci. Technol..

[38] Ekin JW (1984). Strain effects in superconducting compounds. Adv. Cryog. Eng..

[39] Jewell MC, Lee PJ, Larbalestier DC (2003). The influence of Nb_3_Sn strand geometry on filament breakage under bend strain as revealed by metallography. Supercond. Sci. Technol..

[40] Miyoshi Y, van Lanen EPA, Dhallé MMJ, Nijhuis A (2009). Distinct voltage-current characteristics of Nb_3_Sn strands with dispersed and collective crack distributions. Supercond. Sci. Technol..

[41] Cheggour N (2010). Influence of Ti and Ta doping on the irreversible strain limit of ternary Nb_3_Sn superconducting wires made by the restacked-rod process. Supercond. Sci. Technol..

[42] Goodrich LF (2011). Method for determining the irreversible strain limit of Nb_3_Sn wires. Supercond. Sci. Technol..

[43] Cheggour N (2014). Influence of the heat-treatment conditions, microchemistry, and microstructure on the irreversible strain limit of a selection of Ti-doped internal-tin Nb_3_Sn ITER wires. Supercond. Sci. Technol..

[44] Dylla MT, Schultz SE, Jewell MC (2016). Fracture strength distribution of individual Nb_3_Sn filaments. IEEE Trans. Appl. Supercond..

[45] Barth C (2018). Quantitative correlation between the void morphology of niobium-tin wires and their irreversible critical current degradation upon mechanical loading. Scientific Reports.

[46] Cheggour N (2018). Precipitous change of the irreversible strain limit with heat-treatment temperature in Nb_3_Sn wires made by the restacked-rod process. Scientific Reports.

[47] Bajas H (2013). Cold test results of the LARP HQ Nb_3_Sn quadrupole magnet at 1.9 K. IEEE Trans. Appl. Supercond..

[48] Ferracin P (2014). Magnet design of the 150 mm aperture low-*β* quadrupoles for the high luminosity LHC. IEEE Trans. Appl. Supercond..

[49] Ferracin P (2016). Development of MQXF: the Nb_3_Sn low-*β* quadrupole for the HiLumi LHC. IEEE Trans. Appl. Supercond..

[50] Izquierdo S (2016). Second-generation coil design of the Nb_3_Sn low-*β* quadrupole for the high luminosity LHC. IEEE Trans. Appl. Supercond..

[51] Stoynev S (2017). Quench performance and field quality of FNAL twin-aperture 11 T Nb_3_Sn dipole model for LHC upgrades. IEEE Trans. Appl. Supercond..

[52] Stoynev S (2018). Summary of test results of MQXFS1—the first short model 150 mm aperture Nb_3_Sn quadrupole for the high-luminosity LHC upgrade. IEEE Trans. Appl. Supercond..

[53] Santini, C. Ambrosio, G. & Appollinari, G. *MQXFA coils handling & shipping requirements*. US HL-LHC Accelerator Upgrade Project. http://us-hilumi-docdb.fnal.gov/cgi-bin/ShowDocument?docid=820.

[54] Ekin JW (1987). Effect of transverse compressive stress on the critical current and upper critical field of Nb_3_Sn. J. Appl. Phys..

[55] Seeber B, Ferreira A, Abächerli V, Flükiger R (2007). Critical current of a Nb_3_Sn bronze route conductor under uniaxial tensile and transverse compressive stress. Supercond. Sci. Technol..

[56] Mondonico G (2012). Effect of quasi-hydrostatical radial pressure on *I*_c_ of Nb_3_Sn wires. Supercond. Sci. Technol..

[57] Walters CR, Davidson IM, Tuck GE (1986). Long sample high sensitivity critical current measurements under strain. Cryogenics.

[58] Cheggour N, Hampshire DP (2000). A probe for investigating the effects of temperature, strain, and magnetic field on transport critical currents in superconducting wires and tapes. Rev. Sci. Instrum..

[59] Goodrich LF, Cheggour N, Stauffer TC, Filla BJ, Lu XF (2013). Kiloampere, variable-temperature, critical-current measurements of high-field superconductors. J. Res. NIST.

[60] Joint Committee for Guides in Metrology. *Evaluation of measurement data — guide to the expression of uncertainty in measurement*. BIPM, IEC, IFCC, ILAC, ISO, IUPAC, IUPAP, and OIML, JCGM 100:2008, GUM 1995 with minor corrections, https://www.bipm.org/utils/common/documents/jcgm/JCGM_100_2008_E.pdf (2008a)

[61] Seeber B, Mondonico G, Senatore C (2012). Toward a standard for critical current versus axial strain measurements of Nb_3_Sn. Supercond. Sci. Technol..

[62] Taylor DMJ, Hampshire DP (2005). Properties of helical springs used to measure the axial strain dependence of the critical current density in superconducting wires. Supercond. Sci. Technol..

[63] Ekin J (2005). Compressive pre-strain in high-niobium-fraction Nb_3_Sn superconductors. IEEE Trans. Appl. Supercond..

[64] Buehler E, Levinstein HJ (1965). Effect of tensile stress on the transition temperature and current-carrying capacity of Nb_3_Sn. J. Appl. Phys..

[65] Pong, I. & Ekin, J. ESE Scaling spreadsheet. http://www.researchmeasurements.com/ese-scaling-spreadsheet.

[66] International Standard IEC 61788-4. Superconductivity – Residual resistance ratio measurement – Residual resistance ratio of Nb-Ti and Nb_3_Sn composite superconductors. Edition 4.0, 2016-01.

[67] Matsushita T (2018). Round robin test of residual resistance ratio of Nb_3_Sn composite superconductors. IEEE Trans. Appl. Supercond..

