# Publisher Correction: Implications of the strain irreversibility cliff on the fabrication of particle-accelerator magnets made of restacked-rod-process Nb_3_Sn wires

**DOI:** 10.1038/s41598-019-43071-3

**Published:** 2019-05-03

**Authors:** Najib Cheggour, Theodore C. Stauffer, William Starch, Loren F. Goodrich, Jolene D. Splett

**Affiliations:** 1 Department of Physics, University of Colorado, Boulder, CO 80309 USA; 2 Quantum Electromagnetics Division, National Institute of Standards and Technology, Boulder, CO 80305 USA; 3 Applied Superconductivity Center, National High Magnetic Field Laboratory, Florida State University, Tallahassee, FL 32310 USA; 4 Statistical Engineering Division, National Institute of Standards and Technology, Boulder, CO 80305 USA

Correction to: *Scientific Reports* 10.1038/s41598-019-41817-7, published online 02 April 2019

The publication date was omitted from the original PDF version of this Article. This error has now been corrected in the PDF version; the HTML version was correct from the time of publication.

